# Evaluation of Inapparent Dengue Infections During an Outbreak in Southern China

**DOI:** 10.1371/journal.pntd.0003677

**Published:** 2015-03-31

**Authors:** Tao Wang, Man Wang, Bo Shu, Xue-qin Chen, Le Luo, Jin-yu Wang, Yong-zhuang Cen, Benjamin D. Anderson, Mary M. Merrill, Hunter R. Merrill, Jia-hai Lu

**Affiliations:** 1 Zhongshan Center for Disease Control and Prevention, Zhongshan, China; 2 Zhongshan Institute of the School of Public Health, Sun Yat-sen University, Zhongshan, China; 3 Division of Infectious Diseases, Department of Medicine, Duke University, Durham, North Carolina, United States of America; 4 Department of Environmental & Global Health, College of Public Health & Health Professions, University of Florida, Gainesville, Florida, United States of America; 5 Department of Statistics, College of Liberal Arts & Sciences, University of Florida, Gainesville, Florida, United States of America; 6 School of Public Health, Sun Yat-sen University, Guangzhou, China; Duke-NUS, SINGAPORE

## Abstract

Few studies evaluating inapparent dengue virus (DENV) infections have been conducted in China. In 2013, a large outbreak of DENV occurred in the city of Zhongshan, located in Southern China, which provided an opportunity to assess the clinical spectrum of disease. During the outbreak, an investigation of 887 index case contacts was conducted to evaluate inapparent and symptomatic DENV infections. Post-outbreak, an additional 815 subjects from 4 towns with, and 350 subjects from 2 towns without reported autochthonous DENV transmission, as determined by clinical diagnosis, were evaluated for serological evidence of dengue IgG antibodies. Between July and November 2013, there were 19 imported and 809 autochthonous dengue cases reported in Zhongshan. Of 887 case contacts enrolled during the outbreak, 13 (1.5%) exhibited symptomatic DENV infection, while 28 (3.2%) were inapparent. The overall I:S ratio was 2.2:1 (95% CI: 1.1-4.2:1). Post-outbreak serological data showed that the proportion of DENV IgG antibody detection from the 4 towns with and the 2 towns without reported DENV transmission was 2.7% (95% CI: 1.6%-3.8%) and 0.6% (95% CI: 0-1.4%), respectively. The I:S ratio in the 3 towns where clinical dengue cases were predominately typed as DENV-1 was 11.0:1 (95% CI: 3.7-∞:1). The ratio in the town where DENV-3 was predominately typed was 1.0:1 (95% CI: 0.5-∞:1). In this cross-sectional study, data suggests a high I:S ratio during a documented outbreak in Zhongshan, Southern China. These results have important implications for dengue control, implying that inapparent cases might influence DENV transmission more than previously thought.

## Author Summary

In this report, we evaluated individuals with symptomatic and asymptomatic dengue virus (DENV) infections during a 2013 DENV outbreak in Southern China, as well as performed post-outbreak serological testing for DENV IgG antibodies, to better understand DENV transmission. These findings suggest a high rate of asymptomatic cases, which has important implications for future dengue control.

## Introduction

Dengue is one of the most significant mosquito-borne diseases in the world. During the past three decades, the geographical spread of both the mosquito vectors and viruses have led to the global resurgence of epidemic dengue. The World Health Organization (WHO) has estimated that 3.6 billion people live in dengue-endemic areas and that 50 million dengue infections occur annually, with over 2 million causing dengue hemorrhagic fever (DHF) and 21,000 resulting in death [1]. More recent work, which considers both symptomatic and asymptomatic dengue infection, has estimated the global burden of dengue to be much higher, at 390 million infections per year [2].

The clinical manifestations of dengue virus (DENV) infection can be classified as inapparent, undifferentiated febrile illness, classic dengue fever, or the more severe forms, DHF and dengue shock syndrome (DSS). This clinical disease spectrum becomes very important when developing an appropriate surveillance strategy to detect DENV infections. Particularly, challenges can arise when individuals experience mild or asymptomatic infections, as most surveillance programs could easily miss these subclinical cases. Previous surveys conducted in DENV endemic regions have suggested that asymptomatic cases occur more frequently than symptomatic ones, and that the inapparent-to-symptomatic (I:S) ratio varies greatly [3–10]. Given that detectable viremia has been reported among inapparent cases by RT-PCR and virus isolation [11], and that silent circulation of DENV among humans has also been previously documented [4,12], it is possible that asymptomatic DENV infections could cause new foci of disease or eventually an epidemic in non-endemic regions [13]. Thus, it is critical that we fully understand the epidemiology of inapparent dengue infections in order to better develop control strategies to prevent such events.

The one Chinese study conducted in 2009, during an outbreak of DENV-3, the authors estimated the incidence rate of inapparent DENV infections in rural areas throughout Southeastern China to be 28%, but did not attempt to estimate an I:S ratio [14]. Outside of China, a study was conducted during a 2008–2009 dengue epidemic in Australia, where researchers serologically evaluated blood donors to estimate the I:S ratio for DENV infections, which they determined to be 0.59:1 (range 0.18–1.0) [15]. This ratio was markedly lower than similar studies conducted in other endemic regions [3–10]. In 3 other prospective studies that evaluated travelers in non-endemic regions, the I:S ratios were estimated to be 0.75:1, 1.8:1, and 3.0:1 [16–18]. While there have been multiple of such studies looking at inapparent and symptomatic DENV infection ratios, to our knowledge, no such studies have been conducted in China where DENV is a common viral threat in the southern parts of the country.

Re-emergence of dengue in Mainland China was first reported in 1978. Since then, multiple DENV outbreaks have occurred, primarily in Guangdong Province, Southern China [19]. Given there is currently no available evidence to support the presence of any epidemic foci in Mainland China, most researchers purport that the high prevalence of dengue is due to imported cases [20–22]. However, the impact of inapparent infections on the emergence of DENV transmission may call this hypothesis into question if substantiated with appropriate epidemiological data. Therefore, during the 2013 DENV outbreak in Zhongshan, Guangdong Province, China, we conducted a cross-sectional study in order to better understand the dengue virus infection spectrum and to estimate the I:S ratio.

## Materials and Methods

Study methods were reviewed and approved by the Zhongshan Center for Disease Control and Prevention Institutional Review Board. All study participants provided informed consent. The aims of our study were explained, and written informed consent was obtained from all participants. For children less than 16 years of age, consent from the parent or guardian was obtained.

### Study location

**Fig 1:**
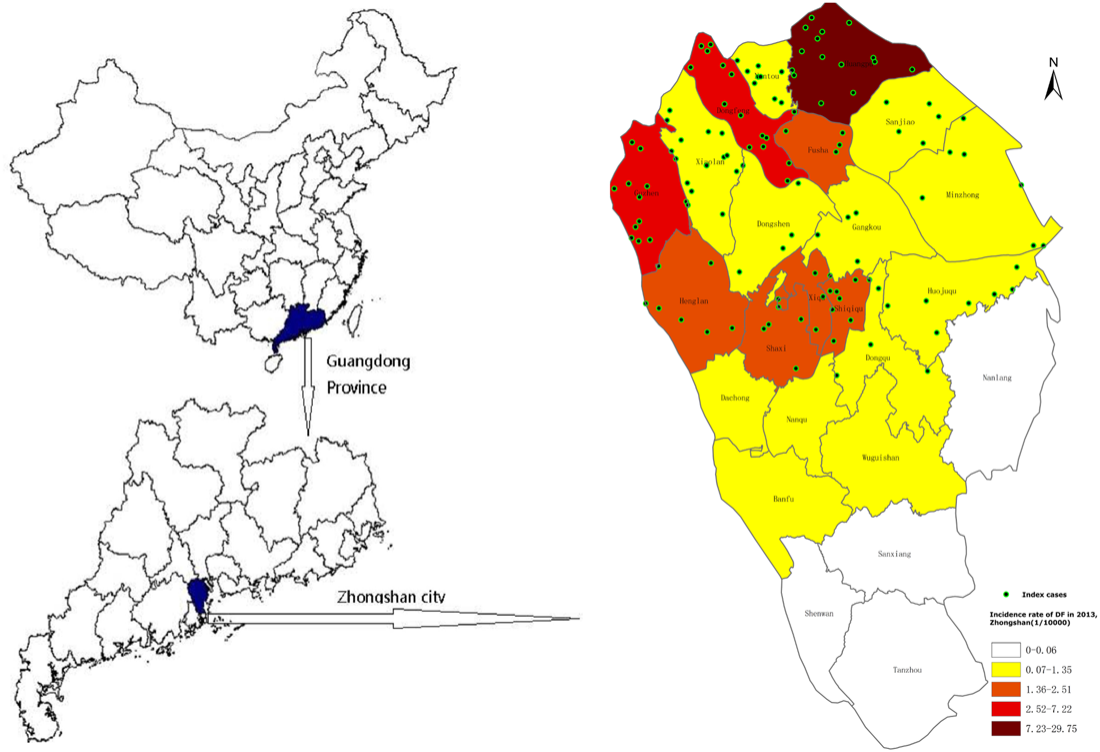
Location and incident rates. The location of Zhongshan, Guangdong Province, China, and a map of the incidence rates of reported dengue cases by town, with the distribution of index cases in 2013.

Zhongshan is located within the Pearl River Delta Region of Guangdong Province, specifically on the west bank of the estuary of the Pearl River. It is geographically connected with Guangzhou (the capital of Guangdong Province) on the north and in vicinity to Hong Kong and Macao. Zhongshan covers an area of 1,800 sq km, consisting of 4 urban and 20 suburban towns (Fig. 1) with a population of 3.15 million. The city is located in a subtropical humid zone with an annual average temperature around 23.0°C and an annual rainfall of 1,791 mm. Vector surveillance data show *Aedes albopictus* to be the predominant mosquito species found throughout the region, which emerge in highest abundance during the rainy season between May and October [23,24]. Since the re-emergence of dengue in 1979, periodic outbreaks, with interepidemic intervals of 2–7 years, have since occurred [25,26]. Sparse detection of dengue IgG antibodies through serological surveillance of healthy individuals and a low number of clinical cases suggest that no outbreaks occurred during 2007–2012 [23].

### Study design

**Fig 2:**
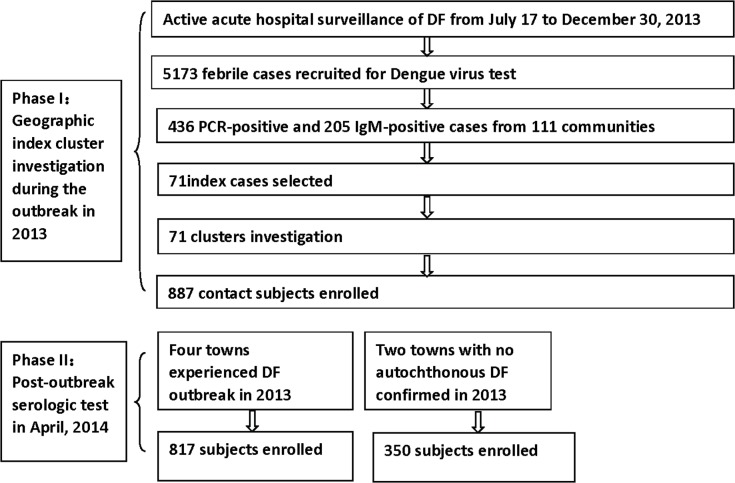
Study design. Overview of the overall study design.

The study was divided into 2 phases (Fig. 2). In phase 1, active dengue surveillance was initiated on July 17, 2013 at select hospitals in Zhongshan. This was after a cluster of 15 dengue laboratory confirmed cases, occurring within 6 days of each other, were reported in Huangpu, which is located in northern Zhongshan. Based on the WHO surveillance recommendations, individuals were screened for DENV if they met a case criterion of acute febrile illness, fever (38°C or above), and at least 2 of the following symptoms: headache, retro-orbital pain, myalgia, arthralgia, rash, flush, hemorrhagic manifestations, leucopenia or thrombocytopenia. If it was within 5 days of fever onset, patients were tested using a commercially available RT-PCR kit (Shanghai ZJ Bio-Tech Co., Ltd.) to identify DENV RNA. After 5 days, a commercial IgM and IgG ELISA kit (Zhongshan Bio-Tech Co., Ltd.) were instead used to detect IgM and IgG antibodies against DENV. According to the Chinese national criteria for dengue diagnosis (WS216-2008), those who were either RT-PCR positive or IgM positive were classified as confirmed dengue cases.

The first confirmed dengue case within a community, without reported travel history to an epidemic focus within 14 days before symptom onset, was eligible to enroll as an index case. Once an index case was identified, local public health workers would visit the area surrounding the home of the index case within 5 days to invite individuals with potential co-exposure to participate in the study. A co-exposure was defined as any persons living in the same household as the index case, or a neighbor living within 100 meters of the index case’s home. Upon enrollment, participants were consented and administered a questionnaire to evaluate if they had experienced any dengue-like symptoms in the previous 30 days. Axilla-temperature was then recorded and 5ml of blood collected. Samples collected from individuals who reported no dengue-like symptoms were then tested for IgM and IgG antibodies against DENV by ELISA (Zhongshan Bio-tech) and samples collected from those who did report dengue-like symptoms were tested for dengue viral RNA using RT-PCR. Clusters were then stratified into the following categories based upon the household characteristics of the index case: construction site, factory, migrant rental, or newly-built community.

Phase 2 was then conducted in April 2014, right before the usual onset of dengue cases. Six towns were selected in total. Huangpu, Guzhen, Xiaolan, and Dongfeng each had documented transmission in 2013, while the towns of Sanxiang and Tanzhou had no autochthonous confirmed cases the same year. Sequential sampling was conducted in each town among apparently healthy individuals seeking a routine physical at local hospitals. A total of 1,167 participants were recruited in this manner. For each participant, a blood sample was collected and tested for IgM and IgG antibodies against DENV using an ELISA kit (Zhongshan Bio-tech), and a questionnaire was administered to retrospectively evaluate if dengue-like symptoms were experienced between June and November of 2013.

### Definitions

According to the dengue surveillance guide of China, imported dengue cases were defined as confirmed dengue cases with a travel history to dengue endemic countries in the past 14 days. In the cluster study, symptomatic dengue was defined as acute febrile illness plus an IgM or RT-PCR positive, while an inapparent dengue infection was defined as having no febrile illness and a positive dengue test (IgM, or RT-PCR). Dengue infections that were diagnosed by ELISA were further categorized as primary infection if the IgM/IgG ratio was ≥ 1.4 and secondary infection if the ratio was < 1.4. In the follow-up serosurvey, symptomatic infection was defined as individuals who retrospectively reported febrile illness during the outbreak period and had an IgG positive test, while an inapparent DENV infection was defined as having no febrile illness during the outbreak period and an IgG positive dengue test.

### Data analysis

For cluster contacts, the infection rate was calculated using the equation: infection rate = (No. of recent infection / No. of cluster contacts)*100%. The inapparent to symptomatic (I:S) infection ratio was equal to the proportion of inapparent infection divided by the proportion of symptomatic infection for a given subgroup (Ratio_IS_ = P_I_/P_S_; P_I_ = the proportion of inapparent DENV infection; P_S_ = the proportion of symptomatic DENV infection). Confidence intervals (95%) for the I:S infection ratio were calculated using $p_{I}/p_{s}\times exp(\pm 1.96\sqrt{{\frac{1}{n\times p}}_{I}+\frac{1}{n\times p_{s}}})$. Statistical difference was determined if the 95% confidence intervals were not overlapping for the same variable. Statistical difference was determined if the 95% confidence intervals were not overlapping for the same variable.

Data for post-outbreak DENV IgG seroprevalence among towns, gender, age groups, and the reported incidence rate was analyzed. Chi-square testing and Spearman correlation analysis were conducted using SPSS 18.0.

## Results

### Characteristic of the notified cases

**Fig 3:**
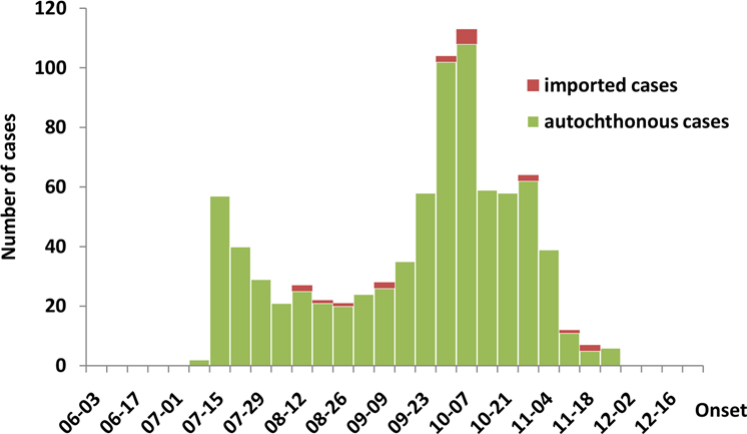
Case distribution. The distribution of dengue cases (n = 828) in Zhongshan, Guangdong Province, China, 2013.

Between July 12^th^ and November 28^th^, 2013 there were 19 imported and 809 autochthonous dengue cases reported to Zhongshan CDC (Fig. 3). During this time, dengue cases peaked twice, once in July when the outbreak was first reported and a second time in October. The reported cases were distributed throughout 108 communities in 22 towns. The incidence rate varied substantially between towns, with the highest rate of 29.7 per 100,000 occurring in Huangpu (Fig. 1). Cases were more frequent among adults aged 50 years or above, while they were less frequent among children 0–14 years of age (Table 1).

**Table 1:** Demographic characteristics and relative risk analysis of 828 reported dengue cases in Zhongshan, Guangdong Province, China, 2013.

Characteristics	No. (%)	Incidence (per 100,000)	Relative Risk (95% CI)
Age, years			
0–14	70 (8)	1.91	1.0
15–49	554 (67)	2.35	1.23 (0.96–1.58)
50–91	204 (25)	4.54	2.38 (1.81–3.12)
Gender			
Female	412 (50)	2.50	1.0
Male	416 (50)	2.77	1.1 (0.96–1.26)

### Evaluation of cluster contacts

Of the total reported dengue cases, 71 were identified and selected as index cases, distributed across 16 towns, with a median age of 33 years, and a similar composition of males and females (Fig. 1). For each cluster, 2–66 contacts (median 8) were enrolled, totaling 887, consisting of 43% females and an average age of 32 years, with 85% aged 15 years or above.

Serological analysis of the 887 cluster contacts showed 41 (4.62%) positive for DENV by either RT-PCR or IgM ELISA, indicating acute or recent dengue infection (95% CI: 3.24%-6.00%). Of these 41, 33 had IgM and IgG test, and only 1 (3.0%) was classified as a secondary infection. Thirteen (1.5%) of the 887 cluster contacts were identified as having a symptomatic DENV infection, while 28 (3.2%) were inapparent. The overall I:S ratio was 2.2:1 (95% CI: 1.1–4.2:1).

Table 2 summarizes the variation of the I:S ratio based on different characteristics of the cluster contacts. Contacts aged 50–91 years had the highest I:S ratio, compared to other age groups, though no significant difference between age groups was found. Three cases were detected among children aged 0–14 years, all of which were symptomatic. A similar I:S ratio was found for both male and female contacts.

**Table 2:** Analysis of dengue infection and inapparent to symptomatic case ratios among cluster contacts by select characteristics in Zhongshan, Guangdong Province, China, 2013.

Characteristics	No. positive/No. tested	Infection rate (%)	Symptomatic infection No. (%)	Inapparent infection No. (%)	Inapparent to symptomatic ratio (95% CI)
Total	41/887	4.6	13 (1.5)	28 (3.2)	2.2:1 (1.1–4.2:1)
Age, y					
<15	3/137	2.2	3 (2.2)	0 (0.0)	0:1 (NA)
15–49	23/169	3.7	7 (1.1)	16 (2.6)	2.3:1 (0.9–5.6:1)
50–91	15/131	11.5	3 (2.3)	12 (9.2)	4.0:1 (1.1–14.2:1)
Gender					
Female	15/380	3.9	5 (1.3)	10 (2.6)	2.0:1 (0.7–5.9:1)
Male	26/507	5.1	8 (1.6)	18 (3.6)	2.2:1 (1.0–5.2:1)
DENV Type					
DENV-1	38/788	4.8	11 (1.4)	27 (3.4)	2.5:1 (1.2–4.9:1)
DENV-3	3/99	3.0	2 (2.0)	1 (1.0)	0.5:1 (0.0–5.5:1)
Household Type					
Construction site	8/97	8.2	3 (3.1)	5 (5.2)	1.7:1 (0.4–7.0:1)
Factory	4/117	3.4	1 (0.9)	3 (2.6)	3.0:1 (0.3–28.8:1)
Migrant rentals	26/569	4.6	7 (1.2)	19 (3.3)	2.7:1 (1.1–6.5:1)
Newly-built community	3/104	2.9	2 (1.9)	1 (1.0)	0.5:1 (0.0–5.5:1)
No. of cluster contacts					
2–10	11/216	5.1	4 (1.9)	7 (3.2)	1.8:1 (0.5–6.0:1)
11–20	18/294	6.1	6 (2.0)	12 (4.1)	2.0:1 (0.8–5.3:1)
21–66	12/337	3.2	3 (0.8)	9 (2.4)	3.0:1 (0.8–11.1:1)

NA: confidence interval not calculated

When stratifying cluster contacts by type of DENV identified in the original index case, contacts of an enrolled index case positive for DENV-1 had a higher I:S ratio compared to contacts of an enrolled index case positive for DENV-3, though this difference was not considered significantly different. When stratified by type of housing, the I:S ratio among contacts from newly-built communities was the lowest (<1). Lastly, higher I:S ratios were associated with higher numbers of contacts in a particular cluster.

### Post-outbreak analysis of DENV IgG antibodies

In April 2014, a total of 1,167 subjects from 6 towns were enrolled and serologically evaluated for IgG antibodies against DENV. The overall proportion of DENV IgG antibodies among subjects from the 4 towns with (Huangpu, Guzhen, Xiaolan, and Dongfeng) reported dengue circulation during 2013 was 2.7% (95% CI: 1.6%-3.8%), while the proportion among subjects from the 2 towns without (Sanxiang and Tanzhou) reported DENV transmission was 0.6% (95% CI: 0–1.4%). Table 3 shows the reported dengue cases, incidence rate, and DENV type from the 2013 outbreak and Table 4 shows the post-outbreak DENV IgG antibody results by participants’ town. Previous exposure (detection of DENV IgG antibodies) was significantly associated with participants’ town (Fisher exact *χ* ^*2*^ = 15.24, p<0.01) and age (p = 0.02). Based on the Spearman correlation analysis, the proportion of DENV IgG antibody detection by town was positively correlated with the reported incidence rate (r = 0.88, p = 0.02). Subjects from Huangpu and Dongfeng had the highest proportion of DENV IgG antibody detection, while those from Sanxiang and Tanzhou had the lowest. The proportion of DENV IgG antibody detection among female subjects from Huangpu was significantly higher than that of males, while the proportion among males and females from Dongfeng were the same. The highest proportion of age-specific DENV IgG antibody detection among subjects from Huangpu and Dongfeng were in the 0–14 year age group. Additionally, there was no increasing trend of DENV IgG antibody detection by age, suggesting no cumulative exposure.

**Table 3:** The 2013 reported dengue cases, incidence rates, and DENV virus types of 6 select towns in Zhongshan, Guangdong province, China.

Town	Reported cases	Incidence rate (per 100,000)	Virus type
Huangpu	434	29.74	DENV-1
Dongfeng	55	4.42	DENV-3
Guzhen	107	7.21	DENV-1
Xiaolan	34	1.07	DENV-1
Sanxiang	1	0.05	DENV-1
Tanzhou	1	0.05	DENV-1
Total	632	5.45	DENV-1, DENV-3

**Table 4:** Post-outbreak prevalence of DENV IgG antibodies among the study population by town, Zhongshan, Guangdong province, China.

		IgG positivity during 2014 testing											
Town	No. positive/No. tested (%)	Disease presentation			Gender (%)				Age (years) (%)				
		Symptomatic infectionin 2013	Inapparent infection in 2013	Infection prior to 2013	Female	Male	*χ* ^*2*^	P-value	0–14	15–49	50–90	*χ* ^*2*^	P-value
Huangpu	11/205 (5.4)	1	9	1	10 (8.7)	1 (1.1)	6.81	0.03	5 (12.5)	4 (3.9)	2 (3.2)	3.44	0.08
Dongfeng	7/212 (3.3)	3	3	1	4 (3.3)	3 (3.3)	0.00	1.00	1 (9.1)	2 (1.5)	4 (6.3)	5.04	0.07
Guzhen	2/200 (1.0)	0	1	1	0 (0.0)	2 (1.8)	1.69	0.50	0 (0.0)	2 (1.6)	0 (0.0)	0.65	1.00
Xiaolan	2/200 (1.0)	0	1	1	0 (0.0)	2 (1.3)	0.51	1.00	0 (0.0)	1 (0.6)	1 (2.5)	4.38	0.37
Sanxiang	1/150 (0.7)	0	0	1	0 (0.0)	1 (1.1)	0.71	1.00	—	1 (0.8)	0 (0.0)	0.25	1.00
Tanzhou	1/200 (0.5)	0	0	1	1 (1.0)	0 (0.0)	0.91	1.00	0 (0.0)	1 (0.7)	0 (0.0)	1.35	1.00
Total	24/1167 (2.1)	4	14	6	15 (2.8)	9 (1.4)	2.78	0.10	6 (5.1)	11 (1.4)	7 (2.7)	7.14	0.02

Of the 24 subjects who were positive for DENV IgG antibodies, none reported having dengue prior to 2013. While four subjects did report having acute febrile symptoms with headache between July and November 2013, only one met the WHO dengue case definition (described above) and was classified as a confirmed case. Assuming the baseline proportion of DENV IgG antibody detection by town was the same as Sanxiang and Tanzhou prior to 2013, the estimated dengue infection I:S ratio during the outbreak in Huangpu, Guzhen and Xiaolan, where DENV-1 circulated, was 11.0:1 (95% CI: 3.7-∞:1), and 1.0:1 (95% CI: 0.5-∞:1) in Dongfeng where DENV-3 circulated.

## Discussion

Prospective cohort studies are a common method for investigating the natural history of dengue infection in endemic regions [3–7,9]. However, it is often not feasible to conduct such studies in non-endemic regions, as it can be difficult to anticipate when and where an outbreak might occur. In this report, we closely investigated index clusters during a DENV outbreak, as well as conducted post-outbreak serological testing for DENV IgG antibodies This allowed us to better understand dengue transmission and to estimate the I:S ratio in a non-endemic city in Southern China. Study data show that adults accounted for the majority of the reported dengue cases and that individuals aged 50 years or more had the highest incidence rate. Additionally, serological analyses following the outbreak show that subjects from Sanxiang and Tanzhou, where no autochthonous dengue was confirmed in 2013, had the lowest proportion of DENV IgG antibody detection.

Previously conducted prospective cohort studies have often shown higher rates of inapparent versus symptomatic infections, with I:S ratios varying by geographic area, epidemiologic context, immunological status of patients, and types of circulating DENV [3–7,9,16,17]. Though our sample size was limited, our investigation does suggest that communities where DENV-3 primarily circulates have a lower I:S ratio than those where DENV-1 is the predominant virus type, which is consistent with a study conducted in Kamphageng Phet, Thailand [5]. Our investigation also suggests a higher I:S ratio among index clusters compared to those using similar study designs conducted in endemic regions [8,10,11,27]. This could be due to differences in DENV vector and host immunity. In contrast to *Aedes aegypti* found in endemic regions, *Aedes albopictus* is the only known vector for dengue in Zhongshan [23], and is thought to be a more susceptible vector of DENV with potential to transmit at low titers resulting in less clinically overt or severe disease [28]. Such was found by Gubler et al. from a comparative analysis of two dengue outbreaks caused by the same serotype. Patients with low levels of viremia were more commonly associated with the outbreak where dengue transmission was less explosive and clinical outcomes less severe [29]. Study data also show that the majority of individuals enrolled in this study experienced a primary DENV infection. When compared to other studies where the majority of the study population had secondary infections, the I:S ratio is markedly higher [3,10,27].

Post-outbreak serological data demonstrates a higher I:S ratio in towns where DENV-1 was the predominant circulating virus type, which was in stark contrast to what was found during the cluster investigation. This outcome could be due to differences in the sampling methods, which introduces the potential for recall bias as some mild febrile cases in the index cluster investigation were classified as inapparent infections. Yoon et al. found similar results in I:S ratios when comparing a prospective cohort with outcomes of the index cluster investigation [10].

It was interesting that no increasing trend of DENV IgG antibody detection by age was found, suggesting no cumulative exposure and that the majority of infections occurred during the 2013 outbreak. This finding is consistent with a previous study conducted by Zhongshan CDC showing sparse detection of DENV IgG antibodies between 2007 and 2012 [23]. The proportions of DENV IgG antibody detection among the 4 towns with confirmed dengue transmission were 7–64 times higher than the incidence rate calculated just from originally reported cases. Coupled with the I:S ratio estimates, it seems likely that a large number of inapparent infections and subclinical cases occurred during the outbreak, which could greatly influence the transmission dynamics of DENV in these areas [30,31].

There were several limitations worth noting. During the index cluster investigation, we were unable to perform a second dengue test following the outbreak, which could have resulted in a higher overall infection rate and subsequently a lower I:S ratio. We did not evaluate how distance between a contact and index cluster might influence DENV transmission. Also, estimates of dengue infection risk may have been impacted by some contacts living within 100 meters of the index cases refusing to participate. These refusal rates were estimated to be between 1% and 10% of the total number of possible enrollees. Lastly, some inapparent and symptomatic infections among individuals following the outbreak may have been misclassified due to the recall bias of the retrospective questionnaire and the inability to determine acute infections with an IgG antibody test.

Despite these limitations, to our knowledge, this is the first report of I:S ratios in China, allowing for a better understanding of the role of inapparent infections in driving DENV transmission in non-endemic regions where *Aedes Albopictus*is the only vector.

## Supporting Information

S1 Checklist STROBE checklist for cross-sectional studies. (PDF)
